# Recent Progress of Novel Electrochemiluminescence Nanoprobes and Their Analytical Applications

**DOI:** 10.3389/fchem.2020.626243

**Published:** 2021-02-09

**Authors:** Shiyu Zhang, Yang Liu

**Affiliations:** Department of Chemistry, Beijing Key Laboratory for Analytical Methods and Instrumentation, Key Laboratory of Bioorganic Phosphorus Chemistry and Chemical Biology of Ministry of Education, Tsinghua University, Beijing, China

**Keywords:** electrochemiluminescence, nanomaterials, biosensing, imaging, structure-properties relationship

## Abstract

High-performance nanomaterials have been seen as a new generation of electrochemiluminescence (ECL) probes or emitters for their finely tunable structure and concomitant remarkable properties, guaranteeing the prospective applications in the analysis and diagnosis devices with superior performances. The structure–activity relationships of ECL nanoprobes in nanoscale are presenting milestone in understanding of the ECL microscopic behaviors and mechanisms, and guide the exploitation of novel ECL probes. In this mini-review, we summarized the recent development of novel ECL probes based on the nanomaterials. The mechanism and relationships between their structure as well as the active sites and functionality were revealed. In addition, the design and regulation of the ECL nanoprobes were emphasized for the biosensing and imaging application. Finally, the potential prospect of the ECL nanoprobes, design, and their applications were discussed.

## Introduction

Electrochemiluminescence (or electrogenerated chemiluminescence, ECL), first reported by Hercules (1964), has become one of the most significant analytical methods till now. Without the interference of exciting light source, ECL has various advantages like low background, high sensitivity, as well as low limit of detection (LOD). Meanwhile, inheriting the merits of electrochemistry, ECL is qualified for the demands such as rapid, *in situ*, and real-time sensing, which are crucial for trace detection, bioassay, and so on. For example, since Leland and Powell (1990) first introduced the ECL emitted by Ru(bpy)_3_^2+^ (bpy = 2,2′-bipyridine) with TPrA (tri-*n*-propylamine) as a co-reactant, it has attached much importance by the fields like clinic diagnosis, and has been one of the most advanced immunoassay systems currently. Evolving the ECL based on Ru(bpy)_3_^2+^ and TPrA, typical ECL immunodiagnostic products like Elecsys^®^ (Roche Diagnostic) are widely used worldwide, taking an irreplaceable part in the analytical chemistry and diagnosis.

Uniformly, the emitters like Ru(bpy)_3_^2+^ which can generate luminescence signals during electrochemistry process are defined as ECL probes, playing the most significant role in ECL procedure. Traditionally, ECL probes can be classified as inorganic probes and organic probes in accordance with their functional component. Inorganic ECL probes, represented by coordination complexes (symbolic example like Ru(bpy)_3_^2+^ and derivatives, and other probes with semblable coordination mode can see Ir(Ⅲ) complexes investigated by Swanick et al. (2012), multicolor complex luminophores studied by Guo et al. (2018)), and usually exhibit tunable wavelength, high efficiency, as well as extreme stability, promising the sensitivity and reliability of analytical requirements. However, such probes are often of expensive cost, and sometimes, the use of heavy metal elements makes them suffer the criticism of biotoxicity. Relatively, organic ECL probes, like luminol and luminol derivatives (Wróblewska et al., 2005; Mayer et al., 2018), porphyrins (Pu et al., 2019; Wu et al., 2020), siloles (Han et al., 2019), and many other kinds, are also of great importance for analysis because of the abundant organic–electronic structures of them. However, traditional ECL probes and systems like those demonstrated above usually depend on bulk reaction, largely limiting their further application in the cases which need the diversity of optical properties. Even worth, it can be found that the design and functional post-modification of them are not always pretty unconstrained.

Fortunately, ECL nanoprobes are firmly believed capable to improve such dilemmas. Recent years, increasing importance are emphasized to the development of high-performance nanomaterials. Extend to the domain of ECL, nanoprobes with smaller size, larger specific surface area, as well as higher surface activity are competent to provide more subtle optical information. Moreover, benefited from advanced synthesis methods as well as rational design and post-modify strategies, ECL nanoprobes possessing controllable morphology, tunable energy level, and component are attached more valuable features like extremer luminescence efficiency, remarkable luminescence intensity, and more flexible adjustability of photophysical properties.

Thanks to the unremitting investigation and evolution of ECL nanoprobes (Chen et al., 2017; Sun et al., 2017; Zhai et al., 2017), the number of exciting breakthroughs in analytical applications keeps adding up. For instance, sensitive bioassay of tumor markers is hopefully becoming more universal (like ECL detection for exosomes, by Zhang et al. (2019)), and spatially resolved ECL imaging can be utilized in smaller scale to analyze electrochemical reactions (like Chen et al. (2020) observed single-particle electrocatalytic activity). Besides, multidimensional motion model of nanoprobes can also be built (Ma et al., 2018) by ECL analysis, which would be incredible and unrealizable without the advantage of ECL nanoprobes.

Exploration, design, and application of ECL nanoprobes are of the essence for chemistry. What follows in this review would sort the novel ECL systems and application cases which got development lately, and related potential prospect would also be summarized.

## Novel System of ECL Nanoprobes

Along with the progress of high-performance nanomaterials’ preparation and investigations, multiple kinds of nanomaterials were found to be able to be promising candidates for high-efficiency ECL nanoprobes. At the meantime, rational design and post-modify strategies of them can usually demonstrate convincing relationships between their structure and ECL activity.

Conventional ECL probes could be an appropriate starting point of this section. Combining with advanced nanotechnologies, traditional emitters are still of crucial value to study. For instance, doping Ru(bpy)_3_^2+^ (or their derivatives) on nanoparticles to react with co-reactant TPrA has been developed as a mature strategy to achieve stable and enhanced ECL for better analytical performance (see Zanut et al., 2020; Cao et al., 2018; *etc*.,). Meanwhile, novel illustration about luminescence pathway can also be found. Like Valenti et al. (2016) stated, the ECL mechanisms of Ru(bpy)_3_^2+^ triethoxysilane derivative (denoted by Rubpy-TES) doped core–shell silica–PEG nanoparticles will swap when the doping level of Ru(bpy)_3_^2+^ changed. Such findings induced well-known systems into nanoprobes, offering persuasive evidence and results to understand ECL behaviors at nanoscale.

**FIGURE 1:**
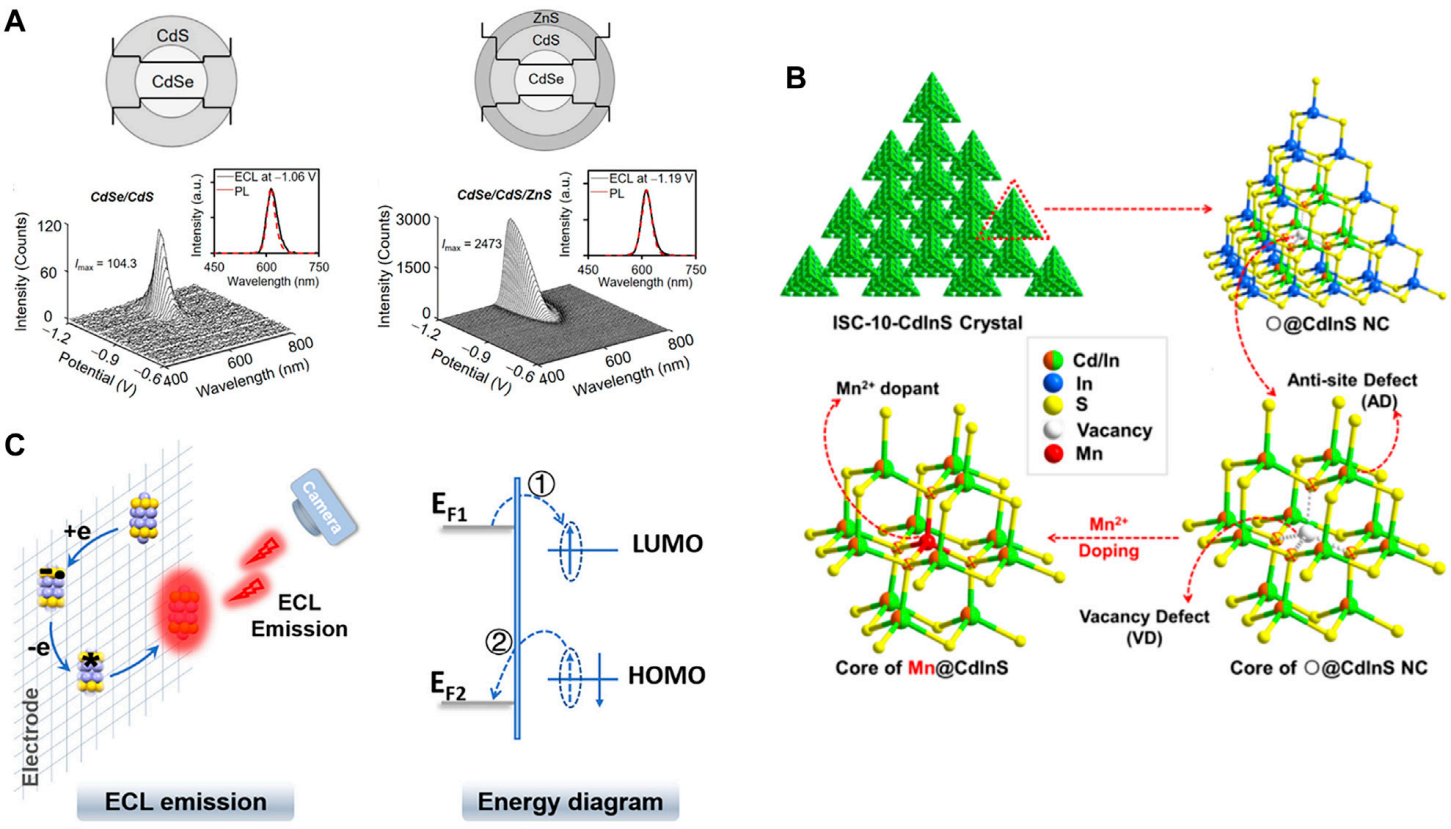
**(A)** ECL spectrum of CdSe/CdS/ZnS core/shell/shell QDs working with S_2_O_8_^2-^. Reproduced from Cao et al. (2020) (https://pubs.acs.org/doi/10.1021/acscentsci.0c00484) with permission. Copyright 2020 American Chemical Society. Further permissions related to the material excerpted should be directed to the ACS.**(B)** The demonstration of vacancy point defect (VD), anti-site defect (AD), and Mn^2+^ precise doping within Cd–In–S ECL nanoprobes. Reproduced from Wang et al. (2016) with permission. Copyright 2016 American Chemical Society. **(C)** The diagram of ECL emission from 25-atom AuAg nanoclusters and corresponding energy transfer. Reproduced from Chen S et al. (2019) with permission. Copyright 2019 American Chemical Society.

Nanomaterials with semiconductor features like nanocrystals, nanoclusters, as well as quantum dots (QDs) can usually present inspiring potential for excellent ECL properties since they possess confinement effect of electron/hole structure, different from bulk semiconductors within which electrons can transfer freely (Ding et al., 2002). Therefore, rational design of semiconductor feature ECL nanoprobes with different photophysical performance can come true if considering the truth that they do have highly tunable energy level and are very sensitive to any tiny changes in surface, defect, or vacancy. A fine example, finished by Cao et al. (2020), showed that by precisely constructing double shell inner inorganic structure (i.e., CdSe/CdS/ZnS as core/shell/shell) and the interface between inorganic and organic component, ultrahigh efficiencies and exceeding narrow band-edge ECL was achieved by QDs working with co-reactant S_2_O_8_^2-^. The additional shell, ZnS, played an essential rule in this QDs ECL system for its much wider bandgap, providing an important protective barrier, thereby guarantying ECL get rid of the influence from surface traps (Figure 1A). The so-resulting ECL can be placed great hope of selective bioassay for its pretty confined spectra width and stable performance.

However, note that the traps (or to say defects) of nanoprobes are not always unfavorable to ECL. Wang et al. (2016) reported an ECL emission generated by intrinsic “vacancy point defect” of a Cd-In-S nanocluster and TPrA (Figure 1B), and realized the recognition between the ECL induced by anti-site defects (AD) and vacancy point defects (VD) *via* wavelength. Besides, precise doping of Mn^2+^ to vacancy point, which can be treated as an efficient method of post-modify, successfully made ECL emission peak red-shift and blocked the VD-induced ECL. This work showed a valuable and reliable design strategy since all the energy-transfer paths followed energy band principles strictly. Additionally, the clear description of nanocrystals, defects, doping cases, and their corresponding ECL behaviors revealed the structure–function relationships distinctly. Following this general idea, AD-assisted enhancement of ECL (Wang et al., 2018b) and mono Cu^+^-induced enhancement of ECL (Wang et al., 2018a) were also achieved, showing the promising future of regulating and applying defects within nanoprobes.

Indeed, structure–function relationships could be a timeless topic full of attraction, especially when the energy diagrams are considered, like the 25-atom AuAg nanoclusters made by Chen S et al. (2019). By digitally controlling the parameters of electrochemistry methods and rational pick of electrochemistry technologies, mechanism of this intense near-IR ECL from 25-atom AuAg nanoclusters was revealed by the electron shift between HOMO-LUMO states (Figure 1C) and 13th Ag atom central position model. Generally, metal nanoclusters often showed excellent luminescence behaviors. So apart from the instance introduced above, many other nanoclusters and novel design ideas can be found, like the Au nanoclusters modified with host–guest assemblage on surface (*via* host–guest recognition) showed efficient ECL with standout monochromaticity (see Yang et al., 2019), and various other examples.

We would also like to emphasize the significance of surface design or surface modification for ECL nanoprobes for their surface-sensitive ECL behaviors. He et al. (2018a) verified that by simply modifying counter ions on the surface of CdTe/CdS nanocrystals, the ECL properties like central wavelength, triggering potential, and intensity would be adjusted, which can be attributed to the tunable surface vacancies which were regulated by the surface-modified counter ions. Analogical work can see He et al. (2018b), ECL performances of ZnSe@CdTe nanocrystals with S_2_O_8_^2-^ as co-reactant were adjusted as well by tunable electron-injection channels, effected by configurational ion-modulated surface states.

Furthermore, some other representative ECL nanoprobes like 2D materials (such as g-C_3_N_4_–decorated Ti_3_C_2_ MXene hybrid nanoprobe by Sun et al. (2020)), single-atom catalysts (single-atom Fe-catalyzed luminol–H_2_O_2_ ECL by Gu et al. (2020)), perovskites (such as CH_3_NH_3_PbBr_3_ perovskite nanocrystals by Tan et al. (2017), Cs_3_Bi_2_Br_9_ perovskite QDs by Cao et al. (2019)), and so on also showed broad application prospect of ECL-based analytical chemistry. The substantial factor can be concluded as fancy structure fabricated by rational design and modify, regulating the energy levels of nanoprobes as well as endowing advanced electronic properties to them. Therefore, the essence of structure–activity relationships can never be too emphasized. Based on them, function-oriented design of nanoprobes can be realized, which benefits the demand-oriented application of ECL.

## Novel Application Based on ECL Nanoprobes

For the past few years, investigations of nanomaterials’ ECL behavior have been boosting the exploring of novel ECL application. A key factor accelerating this trend is outstanding ECL imaging sensitivity brought by high-performance nanoprobes, which can usually be utilized to conduct extremely sensitive biotic analysis and make unpredictable electrochemical mechanisms visualized.

### ECL Biological Analysis and Visualization

Generally, recent years witnessed the revolution of ECL biological analysis, from conventional detection of biomarkers statistically to the observation of single cells or intracellular matters. Literally stating, nanoprobes played a vital role in this process. For instance, Zhang et al. (2019) achieved high-sensitivity diagnostics of exosomes derived from HeLa cells based on the g-C_3_N_4_@galinstan-PDA (polydopamine-coated galinstan liquid metal) shell–core nanohybrid ECL sensors by working with co-reactant S_2_O_8_^2-^.

Although plenty of high-efficiency ECL emitters and strategies were discovered and employed, the urgent pursuing of space-resolved ECL approaches and related probes has never been slowed down. Since Valenti et al. (2017) first reported their pathbreaking work about direct ECL imaging of single cells with spatial resolution based on immunoassay strategy, several further investigations were in hot pursuit. Applying the Ru(bpy)_3_^2+^@SiO_2_/Au nanoparticles—TPrA ECL system, Cao et al. (2018) presented a bipolar immuno-ECL method which can quantitatively detect PSA (prostate specific antigen) in human body fluid samples as well as visually analyze them on the surface of single cells. Another novel instance adopting an unusual way should be that Gao et al. (2020) loaded 12-myristate 13-acetate (PMA) and doxorubicin (DOX) on mesoporous silica nanoparticles (MSN) to get the multifunctional MSN@PMA@DOX hybrids, exhibiting the promising prospect of building composite ECL nanoprobes. Benefiting from the outstanding porous structure of MSN, PMA can be efficiently carried by the hybrids and delivered to HeLa cells, and then stimulating the cells to generate reactive oxygen species (ROS) which reacted with luminol to realize ECL cell imaging. The DOX within hybrids was used to facilitate the apoptosis of HeLa cells.

As demonstrated above, such strategies showed meaningful picture of ECL in the domain of biological analysis, which can be regarded as a profit from recent improvement of nanoprobes on the one hand. On the hand, developing ECL nanoprobes also benefits the visualization of various unpredictable electrochemical mechanisms, for instance, using ECL imaging to track and evaluate electrolytic performance.

### Activity Evaluation of Nanomaterials by ECL Imaging

Considering that Au nanoparticles would improve the generation of ECL from Ru(bpy)_3_^2+^ by enhancing the oxidation of co-reactant TPrA, Pan et al. (2015) first imaged the *in situ* catalytic behaviors of Au nanoparticles, which extraordinarily expanded the application value of nanoprobes’ ECL imaging, leading to the faith in more distinct understanding of electrochemistry at nanoscale. Based on this property, the electrocatalyst screening can be evaluated by ECL techniques.

Following such fundamental logic, Zhu et al. (2018) used ECL imaging to assess electrolytic oxidation performance of metallic and bimetallic nanoparticles like Au, Pt, and Au–Pt Janus nanoparticles, demonstrating that Au–Pt Janus structure had greatly better capacity to catalyze the electrochemical oxidation of Ru(bpy)_3_^2+^ to Ru(bpy)_3_^3+^ than ordinary Au or Pt nanoparticles, which can be certified by the subsequent digital simulation. As described above, the electrochemical redox process of Ru(bpy)_3_^2+^/TPrA system could generate ECL signals. Meanwhile, since nanoparticles were capable to catalyze their transformation, the total ECL intensities calculated from imaging results could straightforwardly mirror their catalytic capacity. However, in order to obtain lower ECL background at this work, the authors coated a thin Al film on the ITO electrodes, which sharply reduced ECL sensitivity, no good for the imaging quality. What is more, only one kind bimetallic probes were investigated by this work, which cannot satisfy current inquiries about increasingly more composite and doping nanostructures.

Hence, based on the pursuing of greater signal to background ratio and better understanding for the catalytic activities of composite nanoparticles, Chen et al. (2020) exploited a novel ECL microscopy technique with superior sensitivity to evaluate electrocatalytic performance of four different nanorods. At this fancy work, lower background signals were achieved by precisely controlling motivating potentials, while the nanorods could catalyze luminol–H_2_O_2_ ECL system to generate high-intensity signals free from interference. Therefore, electrocatalytic performance of four nanostructures could be ranked based on ECL intensities, from which structure dependent electrocatalytic/electrochemical reactivity of those nanoprobes was visualized and demonstrated.

A common ground between the works by Zhu et al. (2018) and Chen et al. (2020) that can be found is that they both use ECL imaging (or the calculated intensities) directly reflecting electrolytic reactivities. At this aspect, Chen M M et al. (2019) intuitively observed inhomogeneous distribution of electrocatalytic reactivity within individual 2D gold nanoplates, which should be attributed to active sites in the electrocatalyst. So far, by the assistance of ECL imaging, it is known that the electrolytic performance varies with not only the structure or component of nanoprobes but also the specific sites.

**FIGURE 2:**
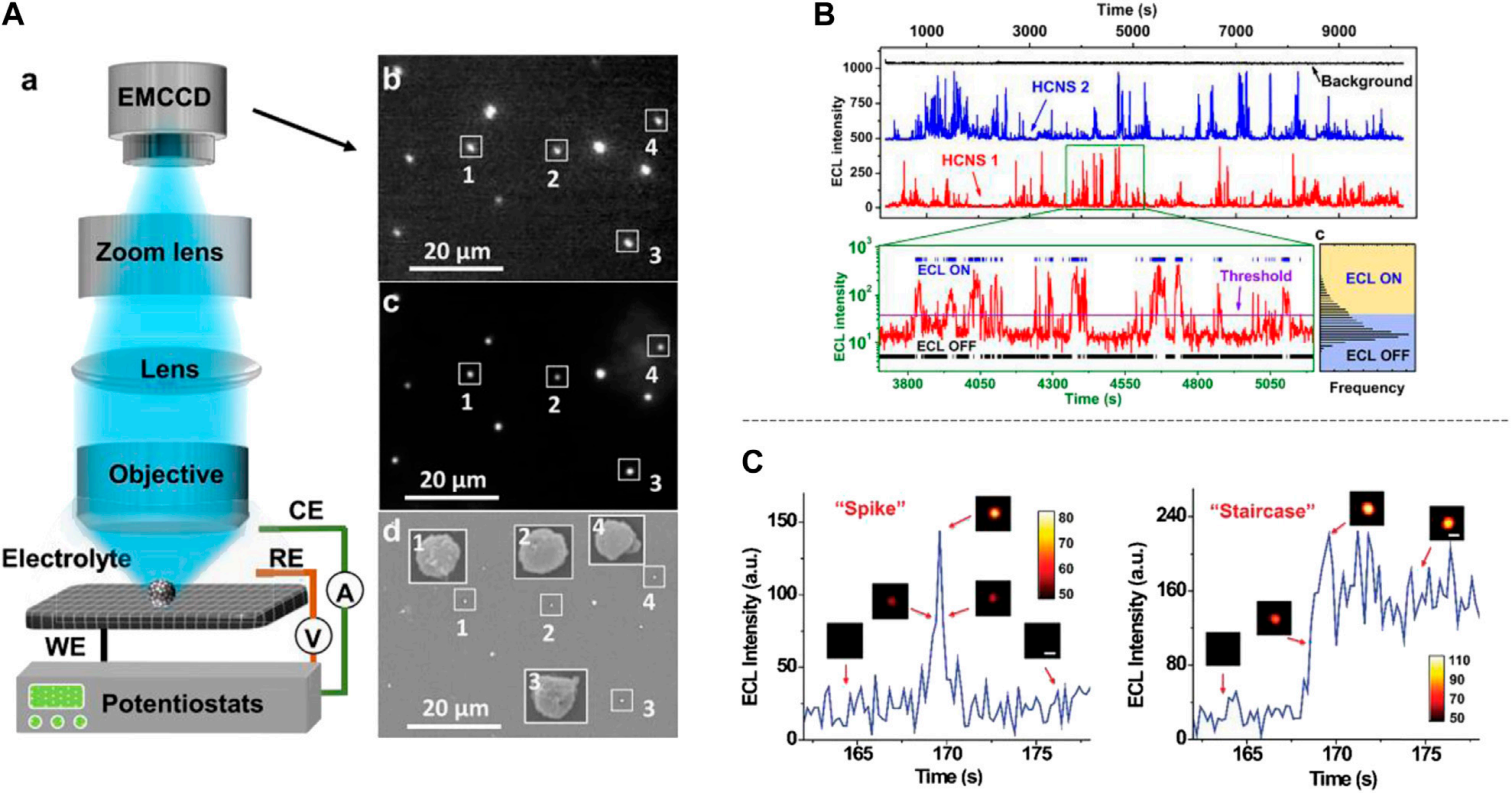
**(A)** General setup of ECL microscope which was used by Ma et al. (2020) to study ECL blinking, **(b)**–**(d)** was ECL image, FL imaging and SEM imaging of HCNSs. **(B)** Two single HCNSs were picked to show the ECL blinking phenomena *via* ECL intensity vs. time graph and the ECL ON/OFF states were stated. **(A)** and **(B)** were reproduced from Ma et al. (2020) with permission. Copyright 2020 American Chemical Society. **(C)** ECL imaging and intensity showed the collision mode of nanoprobes as “spike” or “staircase.” Reproduced from Ma et al. (2018) with permission. Copyright the Royal Society of Chemistry 2018.

To explore the applications of ECL beyond the ECL intrinsic electrochemical system, most recently, Ma et al. (2020) developed a pioneered “ECL blinking” technology to monitor electrochemical hydrogen evolution reaction (HER) at the single particle level (Figure 2A). Different from the “direct observation” strategy described above, indirect ECL methods were explored for the HER evaluation since there is no ECL signal generated by intrinsic HER. To illustrate the relationship between HER activity and ECL “ON/OFF” states (Figure 2B) under ECL microscopy, hollow carbon nitride nanospheres (HCNSs) with advanced electronic structure were used as both of HER catalyst and ECL nanoprobes (with S_2_O_8_^2-^ as co-reactant). Since ECL and HER both happen at the surface of electrodes, the H_2_ bubbles generated from HCNSs during HER affect the ECL signals of HCNSs, and the “ECL blinking” phenomenon was observed and defined. It was found that the ECL blinking behaviors were altering simultaneously with the evolutionary process (generation, growth, and collapse) of H_2_ bubbles during HER. Meanwhile, both the ECL ON-time and OFF-time figured up from two HCNS nanoprobes were of proportional relation with the inverse power–law distributions at probability density dimension. Therefore, it made sense to establish correlation between such ECL imaging behavior and HER activity. Even though some HER catalysts are not able to be ECL emitters, their HER performance can also be evaluated by loading on HCNSs. Hence, this work could enlighten increasingly more electrocatalysts and electrolytic reactions be investigated.

### Multidimensional ECL Analysis

Additionally, for the clearer understanding of electrochemical mechanisms, subtle designed ECL microscopy can also supply efforts to the exploring of stochastic collision electrochemistry. Ma et al. (2018) constructed a set of delicate ECL imaging system to capture the behaviors of single nanoprobes during electrochemistry test. *Via* the optical data originating from the ECL signals of Ru(bpy)_3_^2+^-doping silica nanoparticles (RuDSNs) and TPrA, collision trajectory and collision mode (Figure 2C) of them were (re)established, with the detailed and accurate imaging analysis. By the way, what is worthy to be mentioned is that the movement model of RuDSNs can be described three dimensionally, considering the distance between nanoprobes and electrode surface.

Like introduced above, the impetus trying to build the electrochemical behaviors of nanoprobes at *z*-axis (height) showed the tendency that more dimensions for ECL analysis are essential currently. Considering the features of wave optics, another good attempt to “increase dimensions” is polarized ECL, which was first put forward by Blok et al. (1987), aiming to get the fluctuant information of ECL. Next came to the work by Zinna et al. (2019), which realized polarized ECL by respectively motivating left-hand or corresponding right-hand organic luminophore working with co-reactant benzoyl peroxide (BPO), systematically investigated the asymmetry of enantiomer-based ECL. Nevertheless, to our knowledge, enantio-separation of the chiral ECL probes in such works was complicated to conduct, which may block the universality of polarized ECL. As a consequence, Liang et al. (2020) raised a quite different way to carry out polarized ECL recently. Given the circumstance that surface plasmon coupling (SPC) of nanogranules is polarization angle dependent, the authors combined Au nanoparticles with fluorine-doped BN quantum dots (F-BN QDs), successfully altering the QDs-S_2_O_8_^2-^ triggered isotropic ECL emission to polarized emission. Such polarized ECL systems might not only provide some additional information on the structural symmetry or polarization state of rather than that merely in spatial structure of probes, showing the great prospect of nanoparticles in photophysics of ECL, matching the desire of multidimensional analysis.

## Conclusion and Outlook

In this mini-review, we summarized recent progress about novel ECL nanoprobes based on traditional ECL systems, semiconductor structure probes (QDs, nanoclusters, and nanocrystals), and some others, and emphatically highlighted the idea of “rational design” and “post-modify” to illustrate their structure–activity relationships, trying to provide some guidelines in the future preparation of ECL nanoprobes. Meanwhile, the novel analytical applications of current ECL nanoprobes were revealed by several typical instances, exhibiting the promising application prospect of ECL and nanoprobes.

The future of ECL nanoprobes should still focus on the design of highly efficient and stable luminescence emitters, to satisfy the urgent need of ECL domain like high-resolution space–time imaging and dynamic analysis in complex systems. To meet the requirement, the deep understanding of the ECL mechanism in the novel system should also be emphasized. Moreover, how to develop multidimensional ECL better and make the best use of their photophysical features is worthy of our continuous considering, which will further extend the applications of ECL techniques.
